# Supplementary material for: Potent and broad-spectrum anti-Candida activity of 6α-(3’-methoxy-4’-hydroxybenzoyl)-lup-20(29)-ene-3-one, a triterpenoid from Paullinia pinnata
Source: PLoS One. 2026 Jun 1;21(6):e0350399. doi: 10.1371/journal.pone.0350399 (PMC13225369; doi:10.1371/journal.pone.0350399)
Supplement: S1 Table — SDD: Susceptible dose-dependent. (DOCX) [file pone.0350399.s001.docx]

**S1 Table.** *In vitro* susceptibility patterns of the clinical strains of *Candida* isolates to fluconazole (25 μg), voriconazole (1 µg), miconazole (10 µg), clotrimazole (10 μg), amphotericin B (10 µg) and nystatin (100 units)

| Antifungal drugs | *C. albicans*  n = 11 | *C. glabrata*  n = 13 | *C. tropicalis*  n = 6 | *C. krusei*  n = 5 | *C. parapsilosis*  n = 2 |
| --- | --- | --- | --- | --- | --- |
| Fluconazole | | | | | |
| Resistant | 8 (72.73 %) | 6 (46.15 %) | 3 (50 %) | 5 (100 %) | 1 (50 %) |
| SDD | 2 (18.18 %) | 5 (38.46 %) | 2 (33.33 %) | 0 (0 %) | 1 (50 %) |
| Susceptible | 1 (9.09 %) | 2 (15.38 %) | 1 (16.67 %) | 0 (0 %) | 0 (0 %) |
| Voriconazole | | | | | |
| Resistant | 7 (63.64 %) | 5 (38.46 %) | 2 (33.33 %) | 1 (20 %) | 1 (50 %) |
| SDD | 1 (9.09 %) | 2 (15.38 %) | 1 (16.67 %) | 2 (40 %) | 0 (0 %) |
| Susceptible | 3 (27.27 %) | 6 (46.15 %) | 3 (50 %) | 2 (40 %) | 1 (50 %) |
| Clotrimazole | | | | | |
| Resistant | 8 (72.73 %) | 8 (61.54 %) | 4 (66.66 %) | 2 (40 %) | 1 (50 %) |
| SDD | 3 (27.27 %) | 2 (15.38 %) | 2 (33.33 %) | 2 (40 %) | 1 (50 %) |
| Susceptible | 0 (0 %) | 3 (23.08 %) | 0 (0 %) | 1 (20 %) | 0 (0 %) |
| Miconazole | | | | | |
| Resistant | 6 (54.54 %) | 9 (69.23 %) | 3 (50 %) | 1 (20 %) | 1 (50 %) |
| SDD | 4 (36.36 %) | 3 (23.08 %) | 3 (50 %) | 3 (60 %) | 1 (50 %) |
| Susceptible | 1 (9.09 %) | 1 (7.69 %) | 0 (0 %) | 1 (20 %) | 0 (0 %) |
| Nystatin | | | | | |
| Resistant | 6 (54.54 %) | 3 (23.08 %) | 0 (0 %) | 0 (0 %) | 0 (0 %) |
| SDD | 3 (27.27 %) | 10 (76.92 %) | 6 (100 %) | 3 (60 %) | 1 (50 %) |
| Susceptible | 2 (18.18 %) | 0 (0 %) | 0 (0 %) | 2 (40 %) | 1 (50 %) |
| Amphotericin B | | | | | |
| Resistant | 0 (0 %) | 1 (7.69 %) | 1 (16.67 %) | 0 (0 %) | 0 (0 %) |
| SDD | 4 (36.36 %) | 4 (30.77 %) | 1 (16.67 %) | 1 (20 %) | 0 (0 %) |
| Susceptible | 7 (63.64 %) | 8 (61.54 %) | 4 (66.66 %) | 4 (80 %) | 2 (100 %) |

SDD: Susceptible dose-dependent
