# Supplementary material for: Potent and broad-spectrum anti-Candida activity of 6α-(3’-methoxy-4’-hydroxybenzoyl)-lup-20(29)-ene-3-one, a triterpenoid from Paullinia pinnata
Source: PLoS One. 2026 Jun 1;21(6):e0350399. doi: 10.1371/journal.pone.0350399 (PMC13225369; doi:10.1371/journal.pone.0350399)
Supplement: S3 Table — Values are expressed in µg/mL. MIC: Minimum inhibitory concentration; MFC: Minimum fungicidal concentration; VRC: Voriconazole; KTC: Ketoconazole; AMB: Amphotericin B. Experiment was carried out in duplicate with three technical replicates per treatment and on different days. (DOCX) [file pone.0350399.s003.docx]

**S3 Table**. Antifungal activity of 6α-(3*'*-methoxy-4*'*-hydroxybenzoyl)-lup-20(29)-ene-3-one against the *Candida* strains and isolates

| Specie / Strain | 6α-(3*'*-methoxy-4*'*-hydroxybenzoyl)-lup-20(29)-ene-3-one | | VRC | KTC | AMB |
| --- | --- | --- | --- | --- | --- |
|  | **MIC** | **MFC** | **MIC** | **MIC** | **MIC** |
| *C. albicans* | | | | | |
| ATCC 10231 | 1 | 4 | 8 | 2 | 0.50 |
| Clinical isolate 1 | 1 | 4 | 4 | 1 | 0.50 |
| Clinical isolate 2 | 2 | 8 | 16 | 4 | 1 |
| Clinical isolate 5 | 1 | 2 | 8 | 1 | 0.50 |
| Clinical isolate 9 | 16 | 64 | 32 | 4 | 1 |
| Clinical isolate 11 | 2 | 4 | 8 | 2 | 0.50 |
| Clinical isolate 12 | 16 | 64 | 32 | 8 | 2 |
| Clinical isolate 21 | 1 | 8 | 4 | 1 | 0.50 |
| Clinical isolate 24 | 2 | 8 | 8 | 1 | 0.50 |
| Clinical isolate 31 | 8 | 16 | 16 | 2 | 0.50 |
| Clinical isolate 35 | 4 | 16 | 16 | 2 | 1 |
| Clinical isolate 37 | 4 | 16 | 32 | 4 | 1 |
| *C. glabrata* | | | | | |
| ATCC 2001 | 0.50 | 2 | 4 | 1 | 0.50 |
| Clinical isolate 3 | 0.50 | 2 | 4 | 1 | 0.50 |
| Clinical isolate 7 | 1 | 4 | 8 | 1 | 0.50 |
| Clinical isolate 8 | 2 | 4 | 8 | 2 | 1 |
| Clinical isolate 10 | 1 | 2 | 16 | 2 | 1 |
| Clinical isolate 15 | 1 | 4 | 4 | 1 | 0.50 |
| Clinical isolate 20 | 2 | 4 | 32 | 8 | 2 |
| Clinical isolate 22 | 2 | 4 | 16 | 4 | 1 |
| Clinical isolate 27 | 4 | 16 | 32 | 8 | 2 |
| Clinical isolate 29 | 0.50 | 2 | 8 | 2 | 1 |
| Clinical isolate 32 | 4 | 8 | 32 | 4 | 1 |
| Clinical isolate 33 | 0.50 | 2 | 4 | 1 | 0.50 |
| Clinical isolate 36 | 2 | 4 | 16 | 4 | 1 |
| Clinical isolate 40 | 2 | 8 | 16 | 2 | 1 |
| *C. tropicalis* | | | | | |
| NRRL Y-12968 | 1 | 4 | 8 | 2 | 1 |
| Clinical isolate 6 | 2 | 8 | 8 | 2 | 0.50 |
| Clinical isolate 14 | 1 | 4 | 16 | 4 | 1 |
| Clinical isolate 19 | 1 | 2 | 8 | 2 | 1 |
| Clinical isolate 26 | 4 | 8 | 16 | 4 | 1 |
| Clinical isolate 30 | 1 | 8 | 8 | 2 | 1 |
| Clinical isolate 39 | 8 | 16 | 32 | 8 | 2 |
| *C. krusei* | | | | | |
| ATCC 6258 | 0.50 | 2 | 4 | 1 | 0.50 |
| Clinical isolate 13 | 0.50 | 2 | 4 | 1 | 0.50 |
| Clinical isolate 17 | 1 | 2 | 8 | 2 | 1 |
| Clinical isolate 23 | 1 | 4 | 4 | 1 | 0.50 |
| Clinical isolate 25 | 2 | 8 | 8 | 1 | 0.50 |
| Clinical isolate 28 | 2 | 4 | 8 | 2 | 0.50 |
| *C. parapsilosis* | | | | | |
| ATCC 22019 | 0.50 | 2 | 4 | 1 | 0.50 |
| Clinical isolate 18 | 1 | 2 | 4 | 2 | 0.50 |
| Clinical isolate 38 | 0.50 | 2 | 4 | 1 | 0.50 |

Values are expressed in µg/mL. MIC: Minimum inhibitory concentration; MFC: Minimum fungicidal concentration; VRC: Voriconazole; KTC: Ketoconazole; AMB: Amphotericin B. Experiment was carried out in duplicate with three technical replicates per treatment and on different days.
