# Supplementary material for: Potent and broad-spectrum anti-Candida activity of 6α-(3’-methoxy-4’-hydroxybenzoyl)-lup-20(29)-ene-3-one, a triterpenoid from Paullinia pinnata
Source: PLoS One. 2026 Jun 1;21(6):e0350399. doi: 10.1371/journal.pone.0350399 (PMC13225369; doi:10.1371/journal.pone.0350399)
Supplement: S4 Table — INT: Interpretation, FICI: Fraction Inhibitory Concentration Index. S: Synergism for FICI ≤0.5, I: Indifference FICI was > 0.5 to ≤4.0, and A: Antagonism FICI >4.0. Experiment was carried out in triplicate. (DOCX) [file pone.0350399.s004.docx]

**S4 Table.** FIC indices for the combinations of 6α-(3*'*-methoxy-4*'*-hydroxybenzoyl)-lup-20(29)-ene-3-one with commonly used antifungal drugs against *Candida* strains and clinical isolates

| Specie / Strain | Voriconazole | | Nystatin | | Capsofungin | |
| --- | --- | --- | --- | --- | --- | --- |
|  | **FICI** | **INT** | **FICI** | **INT** | **FICI** | **INT** |
| *C. albicans* | | | | | | |
| ATCC 10231 | 0.50 | S | 0.30 | S | 1.20 | I |
| Clinical isolate 1 | 0.44 | S | 0.40 | S | 1 | I |
| Clinical isolate 2 | 0.28 | S | 0.25 | S | 0.31 | S |
| Clinical isolate 5 | 0.20 | S | 0.38 | S | 1.25 | I |
| Clinical isolate 9 | 0.75 | I | 1.02 | I | 0.75 | I |
| Clinical isolate 11 | 0.38 | S | 0.44 | S | 0.38 | S |
| Clinical isolate 12 | 0.65 | I | 0.28 | S | 0.50 | S |
| Clinical isolate 21 | 0.38 | S | 0.50 | S | 0.35 | S |
| Clinical isolate 24 | 0.25 | S | 0.30 | S | 0.40 | S |
| Clinical isolate 31 | 0.11 | S | 0.50 | S | 0.31 | S |
| Clinical isolate 35 | 0.40 | S | 0.45 | S | 0.44 | S |
| Clinical isolate 37 | 0.75 | I | 0.85 | I | 1.13 | I |
| *C. glabrata* | | | | | | |
| ATCC 2001 | 0.38 | S | 0.40 | S | 0.20 | S |
| Clinical isolate 3 | 0.45 | S | 0.20 | S | 1.12 | I |
| Clinical isolate 7 | 0.28 | S | 0.25 | S | 0.11 | S |
| Clinical isolate 8 | 0.75 | I | 1.02 | I | 0.90 | I |
| Clinical isolate 10 | 0.30 | S | 0.19 | S | 0.25 | S |
| Clinical isolate 15 | 1.24 | I | 1 | I | 1 | I |
| Clinical isolate 20 | 1 | I | 0.90 | I | 1.12 | I |
| Clinical isolate 22 | 0.38 | S | 0.20 | S | 0.28 | S |
| Clinical isolate 27 | 0.50 | S | 0.38 | S | 0.30 | S |
| Clinical isolate 29 | 0.80 | I | 1.02 | I | 0.75 | I |
| Clinical isolate 32 | 0.45 | S | 0.44 | S | 0.19 | S |
| Clinical isolate 33 | 1.13 | I | 0.60 | I | 0.68 | I |
| Clinical isolate 36 | 0.47 | S | 0.77 | I | 1 | I |
| Clinical isolate 40 | 0.35 | S | 0.50 | S | 0.44 | S |
| *C. tropicalis* | | | | | | |
| NRRL Y-12968 | 0.90 | I | 0.75 | I | 0.75 | I |
| Clinical isolate 6 | 0.38 | S | 0.20 | S | 0.28 | S |
| Clinical isolate 14 | 0.44 | S | 0.25 | S | 0.38 | S |
| Clinical isolate 19 | 0.41 | S | 1 | I | 0.61 | I |
| Clinical isolate 26 | 1 | I | 0.80 | I | 0.25 | S |
| Clinical isolate 30 | 0.45 | S | 0.09 | S | 0.40 | S |
| Clinical isolate 39 | 0.61 | I | 1 | I | 0.75 | I |
| *C. krusei* | | | | | | |
| ATCC 6258 | 0.45 | S | 1.13 | I | 1.05 | I |
| Clinical isolate 13 | 0.30 | S | 0.35 | S | 0.25 | S |
| Clinical isolate 17 | 0.28 | S | 0.31 | S | 0.28 | S |
| Clinical isolate 23 | 0.75 | I | 1.24 | I | 0.95 | I |
| Clinical isolate 25 | 0.60 | I | 0.96 | I | 1 | I |
| Clinical isolate 28 | 0.40 | S | 0.40 | S | 0.20 | S |
| *C. parapsilosis* | | | | | | |
| ATCC 22019 | 1.30 | I | 1.13 | I | 0.57 | I |
| Clinical isolate 18 | 0.38 | S | 0.56 | I | 0.40 | S |
| Clinical isolate 38 | 1.50 | I | 1.13 | I | 0.25 | S |

INT: Interpretation, FICI: Fraction Inhibitory Concentration Index. S: Synergism for FICI ≤0.5, I: Indifference FICI was >0.5 to ≤4.0, and A: Antagonism FICI >4.0. Experiment was carried out in triplicate.
