# Supplementary material for: Potent and broad-spectrum anti-Candida activity of 6α-(3’-methoxy-4’-hydroxybenzoyl)-lup-20(29)-ene-3-one, a triterpenoid from Paullinia pinnata
Source: PLoS One. 2026 Jun 1;21(6):e0350399. doi: 10.1371/journal.pone.0350399 (PMC13225369; doi:10.1371/journal.pone.0350399)
Supplement: Attachment — Submitted filename: RESPONSES TO EDITORS QUERIES.docx [file pone.0350399.s012.docx]

DEPARTMENT OF PHARMACOGNOSY AND HERBAL MEDICINE

SCHOOL OF PHARMACY

**RESPONSE TO EDITS REQUESTED: PONE-D-25-59885 - [EMID:94b56f6eecd688ff]**

**EDIT 1**

Please complete all items on the Human Participants Research Checklist that are relevant for your submission, by following this link: https://journals.plos.org/plosone/s/file?id=d3c7/PLOS_Human_Participants_Research_Checklist_2025.pdf (Contact us at plosone@plos.org if you cannot access the document.) There may be overlap between the checklist items and other queries listed below; please address any duplicated queries both in your response email and on the checklist itself. Upload the completed Human Participants Research Checklist as file type “Other” when you re-submit your manuscript. This document is for internal journal use only and will not be published if your article is accepted. The requested information will help us to assess whether your submission complies with PLOS ONE’s policies and adheres to applicable reporting standards. Note that your manuscript may be rejected if you provide incomplete or inadequate responses to the checklist questions and that changing the ‘Section/Category’ of your article does not affect this requirement.

**RESPONSE**

The Human Participants Research Checklist has been completed and included in the submission.

**EDIT 2**

Please provide additional details regarding participant consent. In the ethics statement in the Methods and online submission information, please ensure that you have specified (1) whether consent was informed and (2) what type you obtained (for instance, written or verbal, and if verbal, how it was documented and witnessed). If your study included minors, state whether you obtained consent from parents or guardians. If the need for consent was waived by the ethics committee, please include this information.

**RESPONSE**

The study did not involve human subjects. Therefore, no participant consent was required. We have included this information in Section 2.8: Ethical statement

**EDIT 3**

If you are reporting a retrospective study of medical records or archived samples, please ensure that you have discussed whether all data were fully anonymized before you accessed them and/or whether the IRB or ethics committee waived the requirement for informed consent. If patients provided informed written consent to have data from their medical records used in research, please include this information.

**RESPONSE**

The study used anonymized clinical isolates collected previously during routine diagnostic procedures, with no patient-identifiable information involved. Thus, no consent was required from patients according to the policies of the Ho Teaching Hospital.
