# Supplementary material for: Potent and broad-spectrum anti-Candida activity of 6α-(3’-methoxy-4’-hydroxybenzoyl)-lup-20(29)-ene-3-one, a triterpenoid from Paullinia pinnata
Source: PLoS One. 2026 Jun 1;21(6):e0350399. doi: 10.1371/journal.pone.0350399 (PMC13225369; doi:10.1371/journal.pone.0350399)
Supplement: Attachment — Submitted filename: Objet Detailed Reviewer Report.docx [file pone.0350399.s011.docx]

Objet Detailed Reviewer Report – Manuscript PONE-D-25-59885

Dear Academic Editor,

I have carefully reviewed the manuscript entitled:

**“Potent and Broad-spectrum anti-Candida activity of 6α-(3'-methoxy-4'-hydroxybenzoyl)-lup-20(29)-ene-3-one, a triterpenoid from Paullinia pinnata”**

**(Manuscript ID: PONE-D-25-59885)**

My evaluation was conducted strictly in accordance with the PLOS One publication criteria, with a focus on scientific soundness, technical rigor, transparency of reporting, and compliance with ethical and data availability standards.

**Assessment of PLOS One publication criteria**

**Originality and prior publication**

The manuscript reports original experimental research. The antifungal, antibiofilm, and synergistic activities of the isolated triterpenoid are supported by primary experimental data. There is no indication that the results have been published previously or are under consideration elsewhere.

**Experimental design and methodological quality**

The experimental design is appropriate to address the stated objectives. The methodologies used for compound isolation, antifungal susceptibility testing, antibiofilm assays, and combination studies are clearly described and follow established and recognized protocols. Experimental conditions, controls, and replication are adequately reported, allowing reproducibility of the study.

**Statistical analyses**

The statistical analyses applied are appropriate for the type of data generated. Data presentation is clear, and variability measures are reported where relevant. The statistical treatment supports the interpretation of the results and the conclusions drawn.

**Results and interpretation**

The results are presented in a logical and coherent manner. Figures and tables are clear and informative. The interpretation of the findings is appropriate, and the authors avoid overstatement. The discussion places the results within the context of relevant literature while remaining grounded in the data generated by the study.

**Conclusions**

The conclusions are supported by the experimental results and are presented in a balanced manner. Claims remain within the scope of the data, and no unsupported extrapolations are made.

**Clarity and language**

The manuscript is generally well structured, intelligible, and written in standard scientific English. Minor language and stylistic refinements could improve clarity in some sections, but these issues are limited and do not affect the scientific quality or interpretation of the work.

**Ethics and research integrity**

Ethical considerations are appropriately addressed. The study does not involve direct experimentation on human participants or animals. The use of anonymized clinical isolates obtained from a hospital microbiology laboratory is clearly stated and consistent with institutional policies and research integrity standards.

**Reporting standards and data availability**

The manuscript adheres to appropriate reporting guidelines. A clear Data Availability Statement is provided, and the data supporting the findings are available within the manuscript and/or the Supporting Information files, in compliance with PLOS One policy.

**Overall evaluation and recommendation**

In summary, this manuscript meets all PLOS One publication criteria. The study is scientifically sound, methodologically robust, and transparently reported. The few issues identified are minor and relate primarily to language clarity and formatting rather than scientific content.

I therefore recommend acceptance with minor revision, limited to minor language polishing and formatting consistency.

Thank you for the opportunity to review this manuscript.

**Kind regards,**

**Dr. BOKHARI**

**Reviewer**
