# Supplementary material for: Potent and broad-spectrum anti-Candida activity of 6α-(3’-methoxy-4’-hydroxybenzoyl)-lup-20(29)-ene-3-one, a triterpenoid from Paullinia pinnata
Source: PLoS One. 2026 Jun 1;21(6):e0350399. doi: 10.1371/journal.pone.0350399 (PMC13225369; doi:10.1371/journal.pone.0350399)
Supplement: Attachment — Submitted filename: RESPONSES TO REVIEWERS QUERIES.docx [file pone.0350399.s014.docx]

DEPARTMENT OF PHARMACOGNOSY AND HERBAL MEDICINE

SCHOOL OF PHARMACY

**RESPONSE TO EDITOR AND REVIWERS’ COMMRENTS: PONE-D-25-59885 - [EMID:94b56f6eecd688ff]**

**ACADEMIC EDITOR:**

The manuscript addresses an important topic and presents potentially interesting findings. However, based on the reviewers’ evaluations, substantial revisions are required before the manuscript can be considered for publication. The reviewers have raised significant concerns regarding methodological clarity, experimental validation, data interpretation, and overall presentation. In particular, the authors are requested to provide more detailed response to the reviewer's comments and suggestions. Furthermore, the manuscript would benefit from careful revision of the language to improve clarity, consistency, and readability throughout the text.

**Journal Requirements:**

When submitting your revision, we need you to address these additional requirements.

**COMMENT 1**

Please ensure that your manuscript meets PLOS ONE's style requirements, including those for file naming. The PLOS ONE style templates can be found at

https://journals.plos.org/plosone/s/file?id=wjVg/PLOSOne_formatting_sample_main_body.pdf and

<https://journals.plos.org/plosone/s/file?id=ba62/PLOSOne_formatting_sample_title_authors_affiliations.pdf>

**RESPONSE**

We have reviewed the PLOS ONE style templates and have revised the manuscript to comply with the journal’s formatting requirements, including file naming conventions, heading structure, and reference formatting.

**COMMENT 2**

We note that this submission includes NMR spectroscopy data. We would recommend that you include the following information in your methods section or as Supporting Information files:

1) The make/source of the NMR instrument used in your study, as well as the magnetic field strength. For each individual experiment, please also list: the nucleus being measured; the sample concentration; the solvent in which the sample is dissolved and if solvent signal suppression was used; the reference standard and the temperature.

2) A list of the chemical shifts for all compounds characterised by NMR spectroscopy, specifying, where relevant: the chemical shift (δ), the multiplicity and the coupling constants (in Hz), for the appropriate nuclei used for assignment.

3) The full integrated NMR spectrum, clearly labelled with the compound name and chemical structure.

We also strongly encourage authors to provide primary NMR data files, in particular for new compounds which have not been characterised in the existing literature. Authors should provide the acquisition data, FID files and processing parameters for each experiment, clearly labelled with the compound name and identifier, as well as a structure file for each provided dataset. See our list of recommended repositories here: <https://journals.plos.org/plosone/s/recommended-repositories>.

**RESPONSE**

We appreciate this detailed request. The NMR instrument used in this study was a Bruker Avance-500 (500 MHz), operated at 25°C. This information was already partially stated in Section 2.2 (General Procedures) of the manuscript. We have now expanded the NMR methods description to include all requested details: the nucleus measured (¹H and ¹³C), solvent used (CDCl₃), reference standard (tetramethylsilane, TMS), and temperature. Full integrated NMR spectra are provided as Supporting Information files.

**COMMENT 3**

In your Methods section, please provide additional information regarding the permits you obtained for the work. Please ensure you have included the full name of the authority that approved the field site access and, if no permits were required, a brief statement explaining why.

**RESPONSE**

We thank the editor for this reminder. The only field work carried out was the plant collection. The plant material was collected with permission from the local landowner. No collection permits from the Ghana Forestry Commission were required as *Paullinia pinnata* is not a protected or endangered species in Ghana. We have included that in the ethical statement.

We have also addressed ethical considerations appropriately under the ethical statement section which Reviewer 2 has attested to. The study did not involve the use of human subjects, human tissues or organs or animals. The use of anonymized clinical isolates obtained from a hospital microbiology laboratory is clearly stated and consistent with institutional policies and research integrity standards.

**COMMENT 4**

Please include captions for your Supporting Information files at the end of your manuscript, and update any in-text citations to match accordingly. Please see our Supporting Information guidelines for more information: http://journals.plos.org/plosone/s/supporting-information.

If the reviewer comments include a recommendation to cite specific previously published works, please review and evaluate these publications to determine whether they are relevant and should be cited. There is no requirement to cite these works unless the editor has indicated otherwise.

**RESPONSE**

We have added captions for all Supporting Information files at the end of the manuscript and have updated all in-text citations to refer to the correctly labelled Supporting Information items.

**REVIEWER #1:**

The manuscript entitled “Potent and Broad-spectrum anti-Candida activity of 6α-(3′-methoxy-4′-hydroxybenzoyl)-lup-20(29)-ene-3-one, a triterpenoid from Paullinia pinnata” is scientifically sound and well written. The abstract is concise and informative, the methodology is clearly described and technically adequate, and the results support the authors’ conclusions. The study addresses an important area in antifungal drug discovery and contributes valuable data on plant-derived anti-Candida compounds.

The manuscript is generally acceptable for publication; however, the following points require clarification or justification to further strengthen the study.

Specific Comments for Clarification

**COMMENT 1**

The authors extracted *Paullinia pinnata* leaves using only ethyl acetate, a moderately polar solvent. Sample extraction with solvents of varying polarity could potentially reveal additional or more potent bioactive compounds. Why were highly polar (e.g., methanol, water) or nonpolar (e.g., hexane) solvents not evaluated? Please justify this choice or acknowledgment of this limitation is recommended.

**RESPONSE**

We thank the reviewer for this observation. We initially carried out preliminary screening of various solvent extracts (petroleum ether, ethyl acetate and methanol) of the leaves against the reference strains and found that the ethyl acetate extract showed superior anti-*Candida* activity compared to the petroleum ether and methanol extracts (unpublished data).

We have added the following sentences.

Section 2.4 lines 123 – 126.

Preliminary screening against the *Candida* reference strains was carried out with petroleum ether, ethyl acetate and methanol extracts. The results (data not shown) confirmed greater anti-*Candida* activity in the ethyl acetate extract compared to petroleum ether and methanol extracts.

**COMMENT 2**

The manuscript does not clearly indicate which solvent was used to resuspend the dried extract prior to bioassays. Please specify the solvent used to resuspend the extract. This information is important for reproducibility and interpretation of antifungal activity.

**RESPONSE**

The dried ethyl acetate extract and isolated compound were dissolved in dimethyl sulfoxide (DMSO) to prepare stock solutions for bioassays. This information was partially described in the biofilm methods section (where DMSO is mentioned) but was not explicitly stated in the antifungal assay methods section in our quest to summarize the methods because we have described them in previous publications. We have expanded the description of the bioassays to ensure reproducibility.

Section 2.5.3

**COMMENT 3**

No preliminary phytochemical screening was performed. Such analysis could help correlate observed bioactivity with major classes of secondary metabolites (e.g., terpenoids, flavonoids, phenolics, etc). Please explain why this analysis was not included.

**RESPONSE**

The study was designed as a bioassay-guided fractionation and isolation study focused on the identification of anti-*Candida* compounds from *P. pinnata*. Since the primary objective was isolation and characterisation of the bioactive compound a general preliminary phytochemical screening was not central to the study design. A general preliminary phytochemical screening for classes of secondary metabolite is usually not included in publications that report specific isolated bioactive compounds although it is usually carried out as part of the study.

**COMMENT 4**

While MIC values were determined, MFC testing was not performed by subculturing MIC wells onto Sabouraud Dextrose Agar (SDA). Determining MFC would allow differentiation between fungistatic and fungicidal activity. Please justify its omission or discuss this limitation.

**RESPONSE**

We determined the minimum fungicidal concentrations (MFC) of the *P. pinnata* extract and isolated compound as part of the study (Section 2.5.3 lines 194 -196). The results of those determinations are included in the supplementary data (S2 Table and S3 Table). We only employed the MICs of the column fractions to serve as the guide for the isolation (Table 3).

**COMMENT 5**

The manuscript states that SDA was supplemented with chloramphenicol, but the exact concentration or proportion used is not specified. Please indicate the amount (e.g., µg/mL) and briefly explain why chloramphenicol was included (e.g., suppression of bacterial contamination).

**RESPONSE**

We thank the reviewer for noting this omission. Chloramphenicol was added to Sabouraud Dextrose Agar at a concentration of 50 µg/mL to suppress bacterial contamination during fungal culture and isolation procedures. This is a standard microbiological practice when working with clinical isolates which may contain mixed flora. We have added this detail to the Methods section.

Section 2.5.1 line 142.

**COMMENT 6**

The negative control used in the antifungal assays is not clearly defined. If culture medium alone was used, please justify this choice. Ideally, the negative control should include the solvent used to dissolve the extract, to exclude solvent-related effects.

**RESPONSE**

In a quest to summarise the methods we omitted some important details necessary for reproducibility. We have expanded the methods section particularly section 2.5.3 to include all the negative controls used in the experimentation.

**REVIEWER #2:**

**COMMENT**

In "Potent and Broad-spectrum anti-Candida activity of 6α-(3′-methoxy-4′-hydroxybenzoyl)-lup-20(29)-ene-3-one, a triterpenoid from Paullinia pinnata," a triterpenoid compound's antifungal activity against different Candida species is assessed. The topic is current and relevant, particularly in view of the increasing resistance to antifungals and the ongoing search for novel bioactive natural products.

All things considered, the study answers a significant research question and offers information that PLOS ONE readers might find useful. The experimental work appears generally sound, and the manuscript is clearly written. However, several points should be addressed to improve clarity, rigor, and reproducibility.

The suggested points are intended as minor improvements and do not affect the overall technical soundness of the study or the validity of the authors’ conclusions.

**RESPONSE**

We are grateful to the reviewer for his comments. We acknowledge the minor issues raised and have addressed them to improve clarity, rigor and reproducibility.

**REVIEWER #3:**

**OPENING COMMENTS**

This study reports the antifungal and antibiofilm properties of Paullinia pinnata extract and its isolated triterpenoid, 6α-(3'-methoxy-4'-hydroxybenzoyl) lup-20 (29)-ene-3-one, against drug-resistant Candida spp. The findings are novel and relevant, particularly amid rising antifungal resistance. However, several issues require clarification and refinement before the manuscript meets PLOS ONE’s standards for methodological transparency and cautious interpretation. The authors evaluated antifungal activity, synergism with standard antifungals, and biofilm inhibition by the isolated triterpenoid from P. pinnata. The compound showed promising MIC values, antibiofilm effects, and synergistic interactions with voriconazole, nystatin, and caspofungin. The work aligns with PLOS ONE’s focus on technical soundness and contributes new biological activity data for a natural compound. However, overstated mechanistic claims, missing safety data, and certain structural issues should be addressed.

Strengths:

• Novel bioactivity report for an isolated triterpenoid

• Use of both clinical isolates and reference strains

• Broad panel of Candida species, including drug-resistant isolates

• Combination and antibiofilm assays add depth

Primary Weaknesses (High Priority):

• No cytotoxicity testing limits translational relevance

• Mechanistic explanations are speculative without supporting experiments

• Overly long discussion sections obscure key findings

• Lack of imaging or molecular analysis for biofilm data

**RESPONSE**

We are grateful to the reviewer for the thorough assessment of the work highlighting the strengths and weaknesses of the study. We have addressed the weaknesses to the best of our ability to improve the manuscript.

**Major Issues**

**COMMENT 1**

Cytotoxicity and Safety Data Are Missing

The manuscript repeatedly mentions therapeutic potential, but there is no safety assessment.

Recommended edit:

“We acknowledge that cytotoxicity studies were not conducted, and future work will assess mammalian cell toxicity to evaluate safety.”

Without safety data, claims of potential therapeutic development should be softened.

**RESPONSE**

We agree that the absence of cytotoxicity data is a significant limitation that must be acknowledged. We have softened all claims regarding therapeutic potential throughout the manuscript and have explicitly acknowledged the absence of mammalian cytotoxicity data. We have adopted the recommended language suggested by the reviewer and expanded on this in the Discussion and Conclusion.

We had already acknowledged the absence of the cytotoxicity data as a limitation of the study in the discussion (Section 4 Discussion lines 500 - 502) and had indicated that further work on safety should be done to explore the therapeutic protentional of the compound.

**COMMENT 2**

Mechanistic Claims Are Overstated

Several mechanistic explanations (e.g., efflux pump inhibition, matrix disruption, persister cell effects) are speculative.

Recommended edit:

Replace statements such as:

“The compound could exert its activity by disrupting matrix components…”

with

“These mechanisms are hypothetical and require experimental confirmation.”

**RESPONSE**

Indeed, the mechanistic explanations provided in the Discussion were speculative and not by direct experimental evidence in this study. We have revised all such statements throughout the Discussion to make clear that these are hypothetical mechanisms that the compound could have worked by and not conclusions drawn from our data. We have revised those portions of the discussion to reflect them.

Section 4 lines 464 – 467.

**COMMENT 3**

Biofilm Results Need Clearer Interpretation

No imaging (SEM, confocal) or matrix composition assays were performed. Yet conclusions about structural disruption are made.

Recommended edit:

Emphasize that conclusions are based solely on IC₅₀ measurements.

**RESPONSE**

We thank the reviewer for this important point. We acknowledge that our biofilm conclusions were solely based on IC₅₀ values determined by the XTT reduction assay, and that no imaging or matrix composition assays were performed. We acknowledge this as a limitation of the study (Section 4 Discussion lines 502 -503) and we have also emphasized that in the discussion and conclusion with the reviewer’s recommendation.

**COMMENT 4**

Figures and Tables

The graphs require reformatting the bold text to ensure it remains legible; the bold text is difficult to read in Figures 2-5.

**RESPONSE**

The graphs have been reformatted and all bold text unboldened.

**Minor Issues (Improve clarity, but not essential for validity)**

**ISSUE 1**

The introduction is too long and repeats epidemiological data.

Suggest reducing the prevalence discussion to ~3–4 sentences.

**RESPONSE**

We have reduced the epidemiological data to avoid repetition and making the introduction long.

**ISSUE 2**

Taxonomic formatting:

Ensure *Candida* genus and species names are italicized consistently.

**RESPONSE**

A full manuscript review has been carried out to ensure consistent italicisation of all genus and species names. Instances where *Candida* or species names appeared in non-italic format (particularly in table cells and figure captions) have been corrected.

**ISSUE 3**

Long sentences in discussion:

Several paragraphs contain sentences longer than 40 words; breaking them will improve readability.

**RESPONSE**

Sentences approximately 40 words or more have been revised into two or more shorter sentences without altering the scientific content or interpretation.

**ISSUE 4**

Clarify comparisons to standard antifungals:

The manuscript states that activity is “comparable” to ketoconazole/amphotericin B but does not provide a statistical comparison. Consider rephrasing.

**RESPONSE**

We have revised all such statements to use more appropriate descriptive language, such as “similar MIC range” or “within the same range” and have clarified that formal statistical comparison was not performed.

**ISSUE 5**

The abstract is missing a period at the end of the last sentence.

**RESPONSE**

We have included the period.

**ISSUE 6**

Lines should be numbered to help editors reference specific text for feedback.

**RESPONSES**

We have included the line numbering

**ISSUE 7**

It should be clarified how the cells were prepared before each experiment. The authors did a great job of showing how they were isolated, sourced, and characterized, but there's a lack of detail on whether fresh liquid cultures were used or whether the plates were used as the primary source of cells.

**RESPONSE**

We thank the reviewer for raising this important methodological point. Prior to each experiment, *Candida* strains and isolates were retrieved from -80 °C glycerol stocks and sub-cultured on Sabouraud Dextrose Agar (SDA) at 35 °C for 48 h in a humidified chamber before use.

We have added this information to the Methods section.

Section 2.5.1 lines 148 – 150.
