# Supplementary material for: Potent and broad-spectrum anti-Candida activity of 6α-(3’-methoxy-4’-hydroxybenzoyl)-lup-20(29)-ene-3-one, a triterpenoid from Paullinia pinnata
Source: PLoS One. 2026 Jun 1;21(6):e0350399. doi: 10.1371/journal.pone.0350399 (PMC13225369; doi:10.1371/journal.pone.0350399)
Supplement: Attachment — Submitted filename: Reviewer-Report-plos-one.docx [file pone.0350399.s013.docx]

**Reviewer Report**

**General evaluation**

The manuscript entitled “Potent and Broad-spectrum anti-Candida activity of 6α-(3'-methoxy-4'-hydroxybenzoyl)-lup-20(29)-ene-3-one, a triterpenoid from Paullinia pinnata” investigates the antifungal potential of a plant-derived compound isolated from Paullinia pinnata. The study evaluates antifungal activity against several Candida species, including Candida albicans, and also examines antibiofilm activity and potential synergy with conventional antifungal drugs.

The topic is relevant, as antifungal resistance among Candida species is an important clinical concern. The exploration of bioactive compounds from medicinal plants represents an interesting research direction. The manuscript presents promising preliminary results; however, several aspects of the study require clarification and improvement before the manuscript can be considered for publication.

**Major Comments**

**1-Methodological clarity**

The methodology describing the antifungal assays requires more detailed information. The authors should clearly indicate the number of biological and technical replicates, the preparation of fungal inoculum, and the incubation conditions used during the experiments. These details are essential to ensure the reproducibility of the study.

**2-Characterization of the isolated compound**

Although the compound identification is based on spectroscopic analysis, the manuscript should provide more detailed NMR data. Complete spectroscopic information (chemical shifts, multiplicity, coupling constants) should be clearly presented, and full spectra should be included in the supplementary materials.

**3-Statistical analysis**

The statistical treatment of the experimental data is not sufficiently described. The authors should specify the statistical tests used and present the results with appropriate statistical indicators (e.g., standard deviation, p-values).

**4-Cytotoxicity evaluation**

The study reports significant antifungal activity; however, no cytotoxicity evaluation on mammalian cells is presented. This information is important to assess the potential therapeutic relevance of the compound. At minimum, this limitation should be discussed in more detail in the manuscript.

**5-Discussion improvement**

The discussion section should be expanded to better interpret the results. The authors should provide deeper comparisons with previously reported antifungal triterpenoids and discuss possible mechanisms of action of the compound

**Minor Comments**

The manuscript should be carefully revised for English grammar and readability.

Some sentences in the discussion section are too long and could be simplified for better clarity.

Scientific names of fungal species should be consistently italicized throughout the manuscript.

The quality and clarity of some figures and tables should be improved. Figure legends should provide sufficient detail to understand the experiments without referring to the main text.

The conclusions should be slightly moderated since the current results are based only on in vitro experiments.

**Recommendation**

Major Revision

The manuscript contains interesting findings but requires substantial revisions in methodological description, statistical analysis, and discussion before it can be considered for publication.
