# Supplementary material for: Potent and broad-spectrum anti-Candida activity of 6α-(3’-methoxy-4’-hydroxybenzoyl)-lup-20(29)-ene-3-one, a triterpenoid from Paullinia pinnata
Source: PLoS One. 2026 Jun 1;21(6):e0350399. doi: 10.1371/journal.pone.0350399 (PMC13225369; doi:10.1371/journal.pone.0350399)
Supplement: Attachment — Submitted filename: RESPONSES TO REVIEWERS QUERIES R2.docx [file pone.0350399.s015.docx]

DEPARTMENT OF PHARMACOGNOSY AND HERBAL MEDICINE

SCHOOL OF PHARMACY

**RESPONSE TO REVIWERS’ COMMRENTS**

**REVIEWER 2**

**Reviewer Report**

**General evaluation**

The manuscript entitled “Potent and Broad-spectrum anti-Candida activity of 6α-(3'-methoxy-4'-hydroxybenzoyl)-lup-20(29)-ene-3-one, a triterpenoid from *Paullinia pinnata*” investigates the antifungal potential of a plant-derived compound isolated from *Paullinia pinnata*. The study evaluates antifungal activity against several *Candida* species, including *Candida albicans*, and also examines antibiofilm activity and potential synergy with conventional antifungal drugs.

The topic is relevant, as antifungal resistance among *Candida* species is an important clinical concern. The exploration of bioactive compounds from medicinal plants represents an interesting research direction. The manuscript presents promising preliminary results; however, several aspects of the study require clarification and improvement before the manuscript can be considered for publication.

**Major Comments**

**COMMENT 1: Methodological clarity**

The methodology describing the antifungal assays requires more detailed information. The authors should clearly indicate the number of biological and technical replicates, the preparation of fungal inoculum, and the incubation conditions used during the experiments. These details are essential to ensure the reproducibility of the study.

**RESPONSE**

The number of biological and technical replicates for the antifungal assays have been clearly stated for each experiment performed.

Lines 190 – 191

Lines 212 – 213

Lines 253 - 254

Preparation of the fungal inoculum prior to performing each experiment was stated in section 2.5.1 Lines 148 – 150.

We have also expanded on the inoculum preparation in the in vitro antifungal assay

Section 2.5.3 Lines 175 – 180.

The incubation conditions used during the experiments are also provided for each experiment carried out.

**COMMENT 2: Characterization of the isolated compound**

Although the compound identification is based on spectroscopic analysis, the manuscript should provide more detailed NMR data. Complete spectroscopic information (chemical shifts, multiplicity, coupling constants) should be clearly presented, and full spectra should be included in the supplementary materials.

**RESPONSE**

We thank the reviewer for this valid point. NMR spectra (1D: 1H, 13C, DEPT135; 2D: COSY, HSQC, HMBC) were recorded on a Bruker Avance-500 (500 MHz) in CDCl3. The compound was previously characterized by Jackson et al. (2015) [Ref. 31], and our data were in full agreement with the reported literature. We have now added a structured table of key NMR assignments (1H: chemical shifts, multiplicity, coupling constants and 13C shifts) to Section 3.1. Full spectra (S1–S6 Figs.) are included in the supplementary data and are now explicitly cross-referenced in the section.

**COMMENT 3: Statistical analysis**

The statistical treatment of the experimental data is not sufficiently described. The authors should specify the statistical tests used and present the results with appropriate statistical indicators (e.g., standard deviation, p-values).

**RESPONSE**

We acknowledge this and have revised the manuscript accordingly. All statistical analyses were performed using GraphPad Prism version 8.0. For two-group comparisons, we used an unpaired t-test with Welch’s correction and two-tailed p-value determination; significance was set at p < 0.05. Results are expressed as mean ± SD throughout. While these details were stated in figure legends (Figs. 3 and 4) and in Section 2.7, they were not described in a dedicated sub-section. We have now added a Statistical Analysis sub-section (Section 2.8) to the Methods and ensured consistent reporting of statistical indicators across all relevant results.

**COMMENT 4: Cytotoxicity evaluation**

The study reports significant antifungal activity; however, no cytotoxicity evaluation on mammalian cells is presented. This information is important to assess the potential therapeutic relevance of the compound. At minimum, this limitation should be discussed in more detail in the manuscript.

**RESPONSE**

We fully acknowledge this limitation. Cytotoxicity assays could not be performed in the current study due to the unavailability of appropriate cell culture facilities at our institution at the time. We have now discussed this limitation in substantially greater detail in the Discussion. We emphasize that without a therapeutic index, the translational implications of the antifungal activity cannot yet be fully assessed.

**COMMENT 5: Discussion improvement**

The discussion section should be expanded to better interpret the results. The authors should provide deeper comparisons with previously reported antifungal triterpenoids and discuss possible mechanisms of action of the compound

**RESPONSE**

We appreciate this suggestion and have significantly expanded the Discussion. We have added: (1) a comparative analysis positioning the antifungal activity of 6α-(3′-methoxy-4′-hydroxybenzoyl)-lup-20(29)-ene-3-one against other reported antifungal triterpenoids, including betulin, betulinic acid, ursolic acid, and oleanolic acid, citing their MIC values against Candida species from the literature; and (2) an expanded mechanistic discussion that explores possible modes of action based on (a) the structural features of the lupane skeleton, (b) the synergism pattern observed with drugs representing three different antifungal classes (azoles, polyenes, echinocandins), and (c) structural analogues’ known mechanisms. We clearly state that these remain hypotheses requiring direct experimental confirmation.

**Minor Comments**

**COMMENT 1**

The manuscript should be carefully revised for English grammar and readability.

**RESPONSE**

We appreciate this comment. The entire manuscript has been carefully revised for grammar, clarity, and readability. Specific typographical and grammatical errors identified and corrected include: (1) double word "with with" in Section 2.6; (2) "tropicalsis" (a misspelling of "tropicalis") appearing twice in Sections 3.8.1 and 3.8.2; (3) "whiles" replaced with "while" in Section 3.3; (4) a spurious backtick character before "In the study" in the Discussion; and (5) several long, convoluted sentences rephrased for clarity. The manuscript has been reviewed holistically for grammatical accuracy and fluency throughout.

**COMMENT 2**

Some sentences in the discussion section are too long and could be simplified for better clarity.

**RESPONSE**

Long compound sentences, particularly in the paragraphs describing biofilm resistance mechanisms, and the study limitations, have been broken into shorter, more readable statements. Scientific content has been preserved throughout these revisions.

**COMMENT 3**

Scientific names of fungal species should be consistently italicized throughout the manuscript.

**RESPONSE**

Thank you for pointing this out. We have reviewed the entire manuscript and ensured that all scientific names of fungal species (*Candida* and specific epithets such as *C. albicans*, *C. glabrata*, *C. tropicalis*, *C. krusei*, and *C. parapsilosis*) are consistently italicized throughout.

**COMMENT 4**

The quality and clarity of some figures and tables should be improved. Figure legends should provide sufficient detail to understand the experiments without referring to the main text.

**RESPONSE**

All figures have been re-exported at a minimum resolution of 300 DPI suitable for publication. The figure legends for Figs. 2 – 6 have been expanded to be self-contained, now including: a description of what is shown, the number of biological and technical replicates, how data are expressed (mean ± SD), the statistical test applied, and the meaning of all significance symbols and error bars. Table legends have been updated to include complete footnote explanations for all abbreviations used.

**COMMENT 5**

The conclusions should be slightly moderated since the current results are based only on in vitro experiments.

**RESPONSE**

The Conclusion section has been revised to clearly reflect the in vitro nature of the present findings and to ensure that statements regarding therapeutic potential are appropriately qualified. Language suggesting direct translational applicability has been replaced with statements emphasizing the need for further investigation, including cytotoxicity assessment and in vivo validation, before therapeutic conclusions can be drawn.
