# Supplementary material for: Potent and broad-spectrum anti-Candida activity of 6α-(3’-methoxy-4’-hydroxybenzoyl)-lup-20(29)-ene-3-one, a triterpenoid from Paullinia pinnata
Source: PLoS One. 2026 Jun 1;21(6):e0350399. doi: 10.1371/journal.pone.0350399 (PMC13225369; doi:10.1371/journal.pone.0350399)
Supplement: Attachment — Submitted filename: RESPONSES TO REVIEWERS QUERIES R3.docx [file pone.0350399.s016.docx]

DEPARTMENT OF PHARMACOGNOSY AND HERBAL MEDICINE

SCHOOL OF PHARMACY

**RESPONSE TO REVIWERS’ COMMRENTS**

**Journal Requirements:**

**Comment 1**

Please review your reference list to ensure that it is complete and correct. If you have cited papers that have been retracted, please include the rationale for doing so in the manuscript text, or remove these references and replace them with relevant current references. Any changes to the reference list should be mentioned in the rebuttal letter that accompanies your revised manuscript. If you need to cite a retracted article, indicate the article’s retracted status in the References list and also include a citation and full reference for the retraction notice.

**Response**

We thank the journal for this important instruction. We have carried out a thorough review of the entire reference list of the manuscript. Each reference was individually verified against its source to confirm accuracy of author names, article title, journal name, volume, issue, page numbers and year of publication. We confirm that, to the best of our knowledge, none of the references cited in the manuscript have been retracted. No references have been removed or replaced as a result of this check

**Reviewer #2:**

The manuscript presents a well-conducted and relevant study investigating the antifungal and antibiofilm activity of a triterpenoid isolated from Paullinia pinnata. The revised version has adequately addressed the comments raised in the previous round of review and shows clear improvements in methodological transparency, statistical analysis, and overall presentation.

Only minor editorial points remain:

**Comment 1**

Slightly reinforce the statement in the Conclusion regarding the absence of cytotoxicity data

**Response**

The statement on the absence of cytotoxicity data in the Conclusion has been revised. The revised statement more clearly conveys that the absence of mammalian cell toxicity data is a limitation that must be addressed before the therapeutic potential of 6α-(3′-methoxy-4′-hydroxybenzoyl)-lup-20(29)-ene-3-one can be fully assessed.

**Comment 2**

Ensure that mechanistic interpretations are consistently presented as hypotheses

**Response**

The mechanism of action section of the Discussion and the antibiofilm discussion paragraph have been reviewed. Sentences where mechanistic claims were not sufficiently framed as hypotheses have been revised.

**Comment 3**

Add a brief sentence highlighting the relevance of the antibiofilm findings.

A sentence has been added to the antibiofilm discussion paragraph highlighting the therapeutic relevance of the antibiofilm findings. The sentence draws attention to the fact that biofilm formation is a major driver of treatment failure in vulvovaginal candidiasis and that conventional antifungal agents show reduced efficacy against biofilm-associated *Candida* infections.

**Comment 4**

Minor language polishing and improved consistency in figure legends

Response

The manuscript has been re-read and minor language corrections made throughout. All figure legends have also been reviewed and revised for consistency in structure, terminology and level of detail across Figs. 3–6.

Fig. 3 and Fig. 4 legends each report MIC data with mean ± SD, state the number of biological and technical replicates, name the statistical test and correction applied, and define all significance symbols. These were already consistent with one another.

Fig. 5 and Fig. 6 legends each report IC_50_ data, state the number of biological replicates, describe data as mean percentage inhibition ± SD, and state that IC_50_ values were derived by non-linear regression. These were consistent with one another but differed from Figs. 3 and 4 in the absence of explicit mention of the vehicle control concentration used (1% DMSO). This detail has now been added to both Fig. 5 and Fig. 6 legends for completeness and cross-legend consistency.

**Reviewer #3:**

**Comment 1**

The graph axes remain very difficult to read due to the pixelated font; However, the available tiffs seem to absolve that issue and make the data interpretable.

To ensure your figures meet our technical requirements, please review our figure guidelines: https://journals.plos.org/plosone/s/figures

You may also use PLOS’s free figure tool, NAAS, to help you prepare publication quality figures: https://journals.plos.org/plosone/s/figures#loc-tools-for-figure-preparation.

NAAS will assess whether your figures meet our technical requirements by comparing each figure against our figure specifications.

**Response**

The graph axes have been edited and the writings enlarged to make it easy to read and the data interpretable. We have also the figures using NAAS and ensured that they meet technical requirements.
